# Supplementary material for: Missed opportunities in HCV care: Trends in late diagnosis and treatment
Source: JHEP Rep. 2025 Jun 6;7(9):101474. doi: 10.1016/j.jhepr.2025.101474 (PMC12355104; doi:10.1016/j.jhepr.2025.101474)
Supplement: Multimedia component 1 [file mmc1.pdf]

Missed opportunities in HCV care: Trends in late diagnosis and treatment

Shane Tillakeratne, Heather Valerio, Maryam Alavi, Behzad Hajarizadeh, Marianne Martinello, Kathy Petoumenos, Jacob George, Janaki Amin, Gail V. Matthews, Jason Grebely, Sallie-Anne Pearson, Gregory J. Dore

Table of contents

Table S1………………………………………………………………………………2

Table S2………………………………………………………………………………3

Table S3………………………………………………………………………………5

Fig. S1……………………………………………………………………………...6

Table S4………………………………………………………………………………7

Table S5………………………………………………………………………………8

Table S1. Set of relevant ICD-10 codes for decompensated cirrhosis (DC) and hepatocellular carcinoma (HCC) diagnoses

Inferred Diagnosis	ICD-10 Code	First-time hospitalisation discharge diagnosis
Decompensated Cirrhosis (DC)	R18	Ascites
	I85.0	Oesophageal varices
	I98.3	Oesophageal varices with bleeding in diseases classified elsewhere
	K72.1	Chronic hepatic failure
	K72.9	Hepatic failure, unspecified
	K70.4	Alcoholic hepatic failure
	K76.7	Hepatorenal syndrome
Hepatocellular carcinoma (HCC)	C22.0	Liver cell carcinoma

\

Table S2. ICD-10 definitions used to identify injecting drug use-related hospital presentations among all NSW people with an HCV notification and ESLD diagnosis, NSW 2010-2021

ICD-10	Description
A40	Streptococcal sepsis
A41	Other sepsis
A48.0	Other bacterial diseases, not elsewhere classified (gas gangrene)
B37.6	Candidiasis, candida endocarditis
F11	Mental and behavioural disorders due to use of opioids
F13	Mental and behavioural disorders due to sedatives or hypnotics
F14	Mental and behavioural disorders due to use of cocaine
F15	Mental and behavioural disorders due to use of other stimulants, including caffeine
F19	Mental and behavioural disorders due to multiple drug use and use of other psychoactive substances
G06	Intracranial and intraspinal abscess and granuloma
G09	Sequelae of inflammatory disease of central nervous system
I26.9	Pulmonary embolism, pulmonary embolism without mention of acute or pulmonale
I33	Acute and subacute endocarditis
I34	Nonrheumatic mitral valve disorders
I35	Nonrheumatic aortic valve disorders
I36	Nonrheumatic tricuspid valve disorders
I37	Pulmonary valve disorders
I38	Endocarditis, valve unspecified
I39	Endocarditis and heart valve disorders in diseases classified elsewhere
I40.0	Acute myocarditis, infective myocarditis
I80	Phlebitis and thrombophlebitis
K63.0	Other diseases of the intestine, abscess of intestine
K65.0	Peritonitis, acute peritonitis
K75.0	Other inflammatory liver disease, abscess of liver
L02	Cutaneous abscess, furuncle and carbuncle
L03	Cellulitis
L97	Ulcer of lower limb, not elsewhere classified
L98.8	Other disorders of skin and subcutaneous tissue, not elsewhere classified, other specified disorders of skin and subcutaneous tissue
M54.0	Dorsalgia, panniculitis affecting regions of neck and back
M72.6	Fibroblastic disorders, necrotizing fasciitis
M79.3	Other soft tissue disorders, not elsewhere classified (panniculitis, unspecified)
M86	Osteomyelitis
M89.9	Other disorders of bone, disorder of bone, unspecified
N10	Acute tubulo-interstitial nephritis
R02	Gangrene, not elsewhere classified
R57.2	Shock, not elsewhere classified, septic shock
R65.1	Systemic Inflammatory Response Syndrome of infectious origin with organ failure
R65.9	Systemic Inflammatory Response Syndrome, unspecified
R78.1	Finding of opiate drug in blood
R78.2	Finding of cocaine in blood
T38.7	Androgens and anabolic congeners
T40.0	Poisoning by narcotics and psychodysleptics, opium

T40.1	Poisoning by narcotics and psychodysleptics, heroin
T40.2	Poisoning by narcotics and psychodysleptics, other opioids (codeine/morphine)
T40.3	Poisoning by narcotics and psychodysleptics, methadone
T40.4	Poisoning by narcotics and psychodysleptics, other synthetic narcotics (pethidine)
T40.5	Poisoning by narcotics and psychodysleptics, cocaine
T40.6	Poisoning by narcotics and psychodysleptics, other and unspecified narcotics
T40.8	Poisoning by narcotics and psychodysleptics, lysergide (LSD)
T41.2	Poisoning by anaesthetics and therapeutic gases, other and unspecified general anaesthetics
T42.3	Poisoning by antiepileptic, sedative-hypnotic and antiparkinsonism drugs, barbiturates
T42.4	Poisoning by antiepileptic, sedative-hypnotic and antiparkinsonism drugs, benzodiazepines
T42.5	Poisoning by antiepileptic, sedative-hypnotic and antiparkinsonism drugs, mixed antiepileptics, not elsewhere classified
T42.6	Poisoning by antiepileptic, sedative-hypnotic and antiparkinsonism drugs, other antiepileptic and sedative-hypnotic drugs
T42.7	Poisoning by antiepileptic, sedative-hypnotic and antiparkinsonism drugs, antiepileptic and sedative-hypnotic drugs, unspecified
T42.8	Poisoning by antiepileptic, sedative-hypnotic and antiparkinsonism drugs, antiparkinsonism drugs and other central muscle-tone depressants
T43.6	Poisoning by psychotropic drugs, not elsewhere classified, psychostimulants with abuse potential
T43.8	Poisoning by psychotropic drugs, not elsewhere classified, other psychotropic drugs, not elsewhere classified
T43.9	Poisoning by psychotropic drugs, not elsewhere classified, psychotropic drug, unspecified
T50.7	Poisoning by psychotropic drugs, not elsewhere classified, analeptics and opioid receptor antagonists
X41	Accidental poisoning by and exposure to antiepileptic, sedative-hypnotic, antiparkinsonism and psychotropic drugs, not elsewhere classified
X61	Intentional self-poisoning by and exposure to antiepileptic, sedative-hypnotic, antiparkinsonism and psychotropic drugs, not elsewhere classified
Y11	Poisoning by and exposure to antiepileptic, sedative-hypnotic, antiparkinsonism and psychotropic drugs, not elsewhere classified, undetermined intent

Table S3. ICD-10 definitions used to identify alcohol-use disorder hospital presentations among all NSW people with an HCV notification, NSW 2010-2021

Inferred Diagnosis	ICD-10 Code	Alcohol-use disorder-related hospital admission
**Alcohol-use disorder**	E24.4	Alcohol induced Pseudo-Cushing's syndrome
	F10	Mental and behavioural disorders due to use of alcohol
	G31.2	Degeneration of nervous system due to alcohol
	G62.1	Alcoholic polyneuropathy
	I42.6	Alcoholic cardiomyopathy
	G72.1	Alcoholic myopathy
	Z50.2	Alcohol rehabilitation
	Z71.4	Alcohol abuse counselling and surveillance

Fig. S1. Trends in HCV diagnosis timeliness among people with (A) DC and (B) HCC diagnosis, 2010-2021.

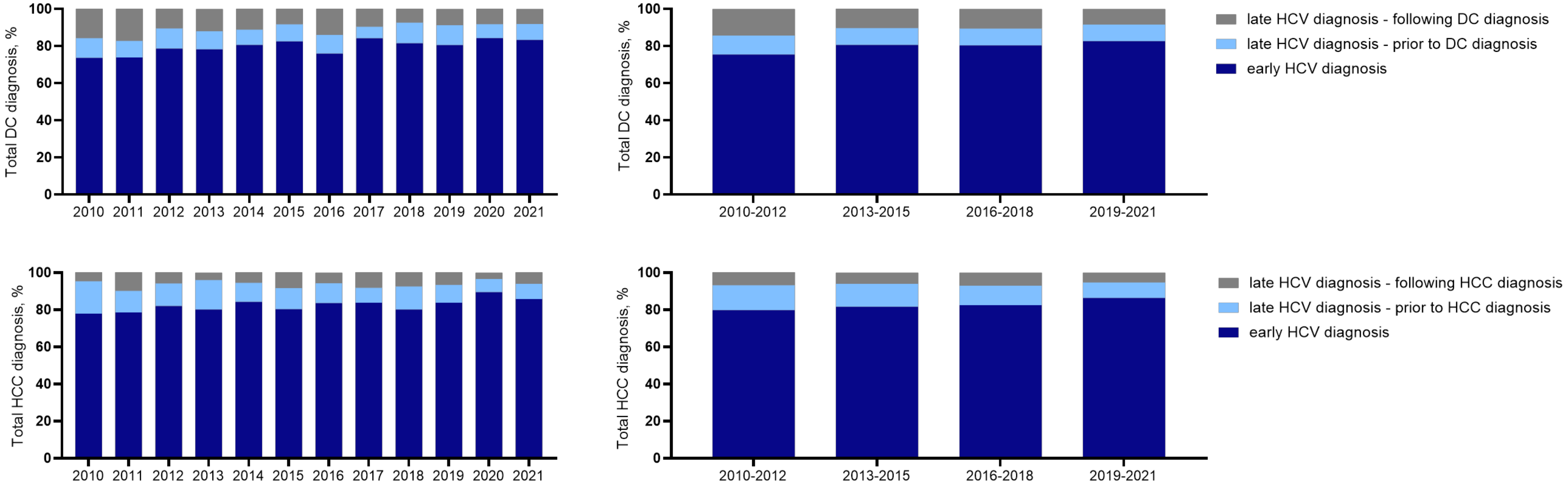

People with an HCV notification and ESLD diagnosis (n = 4,419). (A) People with an DC diagnosis (n = 2,580). (B) People with an HCC diagnosis (n = 1,839). People with an HCV notification and DC/HCC diagnosis (n = 4,419). Late HCV diagnosis – following (within a month or after DC/HCC diagnosis); late HCV diagnosis – prior (0-2 years prior to DC/HCC diagnosis); early HCV diagnosis (>2 years prior to DC/HCC diagnosis). Levels of significance: $p < 0.001$ (Chi-squared test).

Table S4. Proportion of treatment timeliness among people with an HCV diagnosis and ESLD, by time period.

Time period	Total	No Treatment	%	Late treatment	%	Early treatment	%
2010-2021	4,419	2,870	65	1,111	25	438	10
2010-2012	1,081	915	85	148	13	18	2
2013-2015	1,185	837	71	311	26	37	3
2016-2018	1,160	639	55	436	38	85	7
2019-2021	993	479	48	216	22	298	30

Table S5. Demographic characteristics among NSW people with an HCV diagnosis and ESLD by treatment timeliness, 2016-2018 and 2019-2021.

Characteristics, n (row%)	2016-2018						2019-2021					
	No treatment	%	Late treatment	%	Early treatment	%	No treatment	%	Late treatment	%	Early treatment	%
Total, n (row%)	639	55	436	38	85	7	479	48	216	22	298	30
Birth cohort												
≤1944	49	71	19	27	-		36	67	5	9	13	24
1945-1959	268	52	194	38	49	10	185	48	65	17	134	35
1960-1974	269	55	189	39	30	6	217	46	123	26	132	28
≥1975	53	58	34	37	-		37	47	23	29	19	24
Sex [a]												
Male	478	54	344	39	61	7	345	47	169	23	217	30
Female	160	59	92	34	18	7	131	51	47	18	81	31
Region of HCV notification[a]												
Metropolitan	134	51	107	41	23	8	125	55	39	17	60	28
Outer metropolitan	247	60	134	33	29	7	156	48	70	21	101	31
Rural/Regional	244	53	184	40	31	7	183	45	99	24	124	31
Country of birth[a]												
Australia	512	53	383	40	69	7	401	48	182	22	253	30
Overseas	127	65	53	27	16	8	78	50	34	22	45	28
Injecting drug use												
No history	215	53	148	37	40	10	188	49	86	22	113	29
Distant	85	52	69	42	9	6	64	47	25	19	46	34
Recent	339	57	219	37	36	6	227	48	105	22	139	30
Opioid agonist therapy												
No history	468	54	327	38	73	8	370	50	159	17	216	33
Distant	110	57	75	39	7	4	74	45	34	20	58	35
Recent	61	61	34	34	5	5	35	43	23	28	24	29
Alcohol use disorder												
No history	269	56	166	35	45	9	209	47	86	19	152	34
Distant	53	56	32	34	10	10	38	47	11	14	32	39
Recent	317	54	238	41	30	5	232	50	119	26	114	24
Incarceration												
No history	477	55	328	38	65	7	347	50	142	20	215	30
Distant	117	56	78	37	15	7	112	49	56	24	63	27
Recent	45	56	30	38	5	6	20	35	18	31	20	34
Government assistance												
No	60	54	42	38	9	8	45	54	14	17	24	29
Distant	68	46	63	43	17	11	56	43	34	26	40	31
Recent	511	57	331	37	59	6	378	49	168	22	234	29

[a]Among people with available information. HCV, hepatitis C virus; NSW, New South Wales. Small cells (cell count with values <5) were suppressed in this analysis.
