# Supplementary material for: Missed opportunities in HCV care: Trends in late diagnosis and treatment
Source: JHEP Rep. 2025 Jun 6;7(9):101474. doi: 10.1016/j.jhepr.2025.101474 (PMC12355104; doi:10.1016/j.jhepr.2025.101474)
Supplement: Multimedia component 4 [file mmc4.pdf]

JHEP Reports

Missed opportunities in HCV care: Trends in late diagnosis and treatment

Authors

Shane Tillakeratne, Heather Valerio, Maryam Alavi, …, Jason Grebely, Sallie-Anne Pearson, Gregory J. Dore

Correspondence

stillakeratne@kirby.unsw.edu.au (S. Tillakeratne).

Graphical abstract

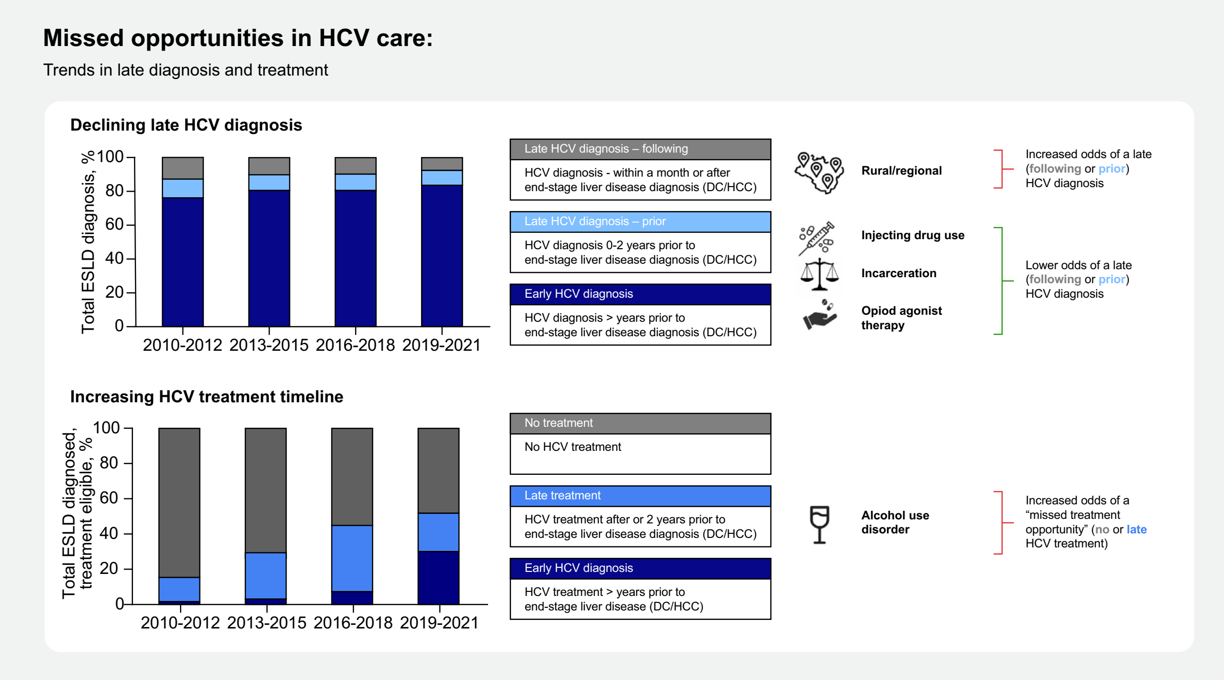

Highlights:

- Timely HCV care essential to prevent liver disease progression.
- Australia has provided universal direct-acting antiviral therapy since 2016.
- Data linkage used to assess population-level HCV diagnosis and treatment timeliness.
- Earlier diagnosis reflects successful targeted screening among key populations.
- Despite progress, gaps remain in rural communities and people with alcohol use disorder.

Impact and implications:

Timely HCV care is essential to prevent liver disease progression. Significant improvements in HCV diagnosis and treatment timing in New South Wales over the past decade highlight the success of Australia's universal provision of direct-acting antiviral therapy and targeted screening initiatives, particularly for people who inject drugs and those recently incarcerated. Persistent barriers to timely care remain for rural communities and people with alcohol use disorder, suggesting the need for enhanced integration of HCV services with alcohol treatment programs and expanded rural outreach. Achieving World Health Organization elimination targets by 2030 requires strengthened efforts to reach underserved populations and better integrate HCV care.

https://doi.org/10.1016/j.jhepr.2025.101474
© 2025 The Author(s). Published by Elsevier B.V. on behalf of European Association for the Study of the Liver (EASL). This is an open access article under the CC BY license (http://creativecommons.org/licenses/by/4.0/).

JHEP|Reports

Missed opportunities in HCV care: Trends in late diagnosis and treatment☆

Shane Tillakeratne[1,*], Heather Valerio[1], Maryam Alavi[1], Behzad Hajarizadeh[1], Marianne Martinello[1], Kathy Petoumenos[1], Jacob George[2,3], Janaki Amin[1,4], Gail V. Matthews[1], Jason Grebely[1], Sallie-Anne Pearson[5], Gregory J. Dore[1]

JHEP Reports **2025**. vol. 7 | 1–12

Check for updates

Background & Aims: Timely HCV care is essential to prevent liver disease progression. The aim of this study was to evaluate trends in late HCV diagnosis and treatment in people diagnosed with end-stage liver disease (ESLD) in New South Wales (NSW), Australia.

Methods: HCV notifications in NSW, Australia (1995-2022) were linked to hospital admissions (2010-2021) and treatment records (2002-2022). Descriptive analyses and logistic regression were used to examine trends and factors associated with late diagnosis and missed treatment opportunities. Late diagnosis and treatment were defined as the absence of HCV notification and treatment within 2 years prior to or following the first hospitalisation for ESLD.

Results: Among 4,419 people with an HCV notification and ESLD diagnosis, late HCV diagnoses decreased from 24% in 2010-2012 to 16% in 2019-2021. The proportion receiving no or late treatment declined from 98% (85% no, 13% late) to 70% (48% no, 22% late). Residing in rural or regional areas was linked with late HCV diagnosis (adjusted odds ratio [aOR] 1.44, 95% CI 1.05-1.97, p = 0.024). Recent injecting drug use (aOR 0.78, 95% CI 0.60-0.99, p = 0.041), incarceration (distant [aOR 0.55, 95% CI 0.38–0.78, p = 0.001], recent [aOR 0.51, 95% CI 0.28–0.96, p = 0.037]), government assistance (aOR 0.57, 95% CI 0.39-0.82, p = 0.002), and older age (born ≤1944 [aOR 0.31, 95% CI 0.15-0.66, p = 0.002], born 1945-1959 [aOR 0.47, 95 CI% 0.29-0.77, p = 0.003]), were associated with lower odds of a late HCV diagnosis. Recent alcohol use disorder was associated with increased odds of no or late treatment (aOR 1.80, 95% CI 1.40-2.32, p = 0.001).

Conclusion: Encouragingly, factors associated with social marginalisation predict earlier HCV diagnosis, while rural/regional residence predicts late HCV diagnosis among people with ESLD. Missed HCV treatment opportunity, defined by no or late treatment is associated with alcohol use disorder, but not with indicators of social marginalisation.

© 2025 The Author(s). Published by Elsevier B.V. on behalf of European Association for the Study of the Liver (EASL). This is an open access article under the CC BY license (http://creativecommons.org/licenses/by/4.0/).

Introduction

The introduction of direct-acting antiviral (DAA) therapy has transformed the clinical management of HCV, providing a cure for the vast majority of those treated..[1] This provided the foundation for World Health Organization's (WHO) 2030 hepatitis C elimination targets to diagnose 90%, treat 80% of those eligible, and reduce liver-related mortality attributable to HCV to ≤2 per 100,000 population.[2] Effective HCV treatment prevents progression to end-stage liver disease (ESLD) and liver-related mortality.[3] Jurisdictions across the globe have demonstrated that publicly funded access to DAA therapy can reduce liver-related morbidity and mortality.[4,5]

Many individuals with chronic HCV infection remain undiagnosed, and among those diagnosed, many fail to access timely care. Consequently, many people living with HCV only enter care after developing advanced liver disease complications, resulting in suboptimal treatment outcomes.[6] We previously developed a definition of "late diagnosis" as hepatitis diagnosis within 2 years or following an ESLD diagnosis – either decompensated cirrhosis (DC) or hepatocellular carcinoma (HCC).[7] We have extended this research to evaluate late HCV treatment, as defined by DAA therapy within 2 years or following an ESLD diagnosis, as well as no treatment. We describe this combined group as having a "missed treatment opportunity", given the potential impact of earlier DAA therapy on liver disease progression and ESLD risk. Examining drivers of late diagnosis and missed treatment opportunity could provide important insights to inform strategies to improve HCV clinical management and optimise DAA utilisation.[8]

Leveraging whole-of-population linked data, the primary aim of this study was to examine trends in late HCV diagnosis and missed treatment opportunity among people diagnosed with ESLD in New South Wales (NSW), Australia. Additionally, as a secondary aim, the study sought to identify factors associated

☆ Given their role as Co-Editors, Jacob George had no involvement in the peer-review of this article and had no access to information regarding its peer-review. Full responsibility for the editorial process for this article was delegated to the Deputy Editor Xavier Forns and Editor-in-Chief Josep Llovet.

* Corresponding author. Address: The Kirby Institute, UNSW Sydney, Sydney, New South Wales, Australia; Tel.: +61-2-9385 0957.
E-mail address: stillakeratne@kirby.unsw.edu.au (S. Tillakeratne).

https://doi.org/10.1016/j.jhepr.2025.101474

with late HCV diagnosis and missed treatment opportunity in this population.

Patients and methods

Setting, data sources and linkage

NSW accounts for approximately 35% of the HCV burden in Australia,[9] offering a unique setting to examine progress in addressing gaps in HCV diagnosis and treatment. Key features that enhance the population-level capacity to evaluate HCV disease outcomes are mandatory HCV notification for more than three decades, standardised collection of hospitalisation admission data, and Australia's universal healthcare including all HCV treatment provided through Federal Government subsidisation.

Several administrative datasets were included in the data linkage: NSW Notifiable Conditions Information System which contains all records of NSW residents with HBV and HCV positive serology dating from 1993; NSW Admitted Patient Data Collection contains all inpatient data from 2002; NSW Registry of Births, Deaths and Marriages includes date of death from 1993; NSW Bureau of Crime Statistics and Research dataset contains all prison reception and discharge dates from 1998; NSW Electronic Recording and Reporting of Controlled Drugs system, for opioid agonist therapy (OAT) authority data from 1985. The NSW Centre for Health Record Linkage conducted probabilistic record linkage using automated and algorithmic techniques to block and score potential matches. This approach combined probabilistic methods with ChoiceMaker software, which applied machine learning algorithms and deterministic rules to link datasets with people notified with HCV. A subsequent probabilistic linkage by the Australian Institute of Health and Welfare, using individual Medicare numbers (Australia's universal healthcare identifiers), linked the Pharmaceutical Benefits Scheme, which contains records of HCV treatment dispensing since 2002, and the DOMINO (Data Over Multiple Individual Occurrences) dataset, which has provided event-based information about welfare recipients from the Australian Department of Social Services since 2002.

Observation period

Data were extracted from each database as follows: HCV notifications (1 January 1993-31 March 2022); hospitalisations (1 July 2001-31 December 2021); date of death (1 January 1993-31 December 2021); OAT (1 January 1985-31 December 2021); incarcerations (1 January 1994-31 December 2021); HCV therapy (1 July 2002 - 31 December 2022); government assistance (1 January 2002 - 31 December 2021).

To examine trends in late HCV diagnosis and late treatment initiation in relation to ESLD, four key time periods were explored to capture the evolving HCV treatment landscape in NSW. These periods reflect the expansion of treatment options: pegylated interferon+ribavirin access period (January 1, 2010 - December 31, 2012); pegylated interferon+ribavirin/DAA period (January 1, 2013 - December 31, 2015); early DAA period (January 1, 2016 - December 31, 2018), and pangenotypic DAA period (January 1, 2019 - December 31, 2021).

Inclusion criteria

All people with an HCV notification recorded in NSW from January 1, 1995, to March 31, 2022, who were diagnosed with ESLD during the study period: January 1, 2010, to 31 December 2021.

Exclusion criteria

HCV notifications occurring before 1995 were excluded to address potential data quality issues and reduced antibody specificity associated with early assays (n = 630). In addition, records were excluded if they had a missing date of birth (n = 1), if the individual was under 18 years of age at the end of the follow-up period (n = 5), or if the record could not be linked to the Pharmaceutical Benefits Scheme via a Medicare number (n = 458).

Study outcomes

The primary outcomes of interest were a) late HCV diagnosis among people with HCV and ESLD diagnosis in NSW, defined as HCV notification occurring within 2 years prior or following first hospitalisation for ESLD (DC or HCC), during 1 January 2010 to 31 December 2021; and b) late HCV treatment initiation among people with HCV and ESLD diagnosis in NSW, defined as HCV treatment occurring within 2 years prior or following first hospitalisation for ESLD, during 1 January 2010 to 31 December 2021, as recorded in the Pharmaceutical Benefits Scheme.

Inpatient hospital records were used to identify ESLD (DC and HCC) diagnoses. Each episode included ICD-10 coding with primary and secondary diagnoses, allowing for a maximum of 50 diagnostic fields. For DC, diagnostic codes that allow for comparison with prior studies were used: ascites (R18), oesophageal varices with bleeding (I85.0 and I98.3), chronic liver failure (K72.1 and K72.9), alcoholic liver failure (K70.4), and hepatorenal syndrome (K76.7), as detailed in Table S1.[7] For HCC, the single diagnostic code used was liver cell carcinoma (C22.0). In cases of dual diagnosis, HCC diagnosis was prioritised.

Population demographics and characteristics

Factors of interest included: birth cohort (≤1944,1945-1959,1960-1974, ≥1975), sex (male, female), region of HCV notification (rural/regional, outer metropolitan, metropolitan), country of birth (Australia, overseas), injecting drug use (no history, distant, recent), OAT (no history, distant, recent), alcohol use disorder (no history, distant, recent), incarceration (no history, distant, recent) and receipt of government assistance (no history, distant, recent). The social marginalisation indicators were chosen specifically to examine populations that face social disadvantage and healthcare barriers in the context of HCV care. A weighted evidence algorithm, based on NSW Health's data linkage strategy, was used to comprehensively identify country of birth information across multiple datasets.[10] Evidence of injecting drug use was characterised based on hospitalisations for injecting drug-related harm.[11] ICD-10 definitions used to identify injecting drug use-related hospital presentations among all NSW people with an HCV notification are provided in Table S2. OAT was defined as evidence of dispensing as determined by the OAT authority's recorded start

and stop dates. Alcohol use disorder is a standard term used to define continued drinking despite adverse mental and physical consequences.[12] A hospital diagnosis code (ICD-10) was used to infer the presence of alcohol use disorder; coded in either the principal or a secondary diagnosis field of a linked inpatient hospital record (Table S3). Government assistance was defined as any welfare payment recorded in the DOMINO dataset.

For the variables injecting drug use, OAT, alcohol use disorder, incarceration, and government assistance, "recent" was defined as any record within 2 years preceding the DC/HCC diagnosis, while "distant" referred to the last observed record occurring prior to this period.

Statistical analysis

Analysis 1: Characteristics of people with HCV notification and ESLD diagnosis in NSW, Australia

Descriptive analyses were conducted to characterise the HCV notified and ESLD diagnosed population between January 1, 2010, and 31 December 2021, by the covariates of interest, stratified by time period.

Analysis 2: Proportions of late HCV diagnosis and no or late HCV treatment among people with HCV notification and ESLD diagnosis, 2010-2021

Proportions of late HCV diagnosis and no or late HCV treatment among people with HCV notification and ESLD diagnosis were calculated using the same underlying population of people in Analysis 1. Proportions were stratified by year of ESLD diagnosis and time period, and cohort characteristics were also tabulated.

Building upon the previously defined late diagnosis criteria (HCV notification occurring within 2 years prior or following ESLD diagnosis), people with an ESLD (DC or HCC) diagnosis were further stratified into three detailed categories based on the temporal relationship between HCV and the DC/HCC diagnosis: a) early diagnosis (>2 years prior to DC/HCC diagnosis); b) late diagnosis – prior (0-2 years prior to DC/HCC diagnosis); c) late diagnosis – following (within a month or after DC/HCC diagnosis). For this analysis, categories b and c were considered as late diagnoses.

To assess HCV treatment timing, people with an ESLD (DC or HCC) diagnosis were stratified into three categories according to the time between their HCV treatment and DC/HCC diagnosis: a) early treatment (>2 years prior to DC/HCC diagnosis); b) late treatment (after or within 2 years prior to ESLD diagnosis); and c) no treatment. Chi-squared test was used to evaluate temporal changes in the proportion of people with late HCV notification and no or late HCV treatment.

Analysis 3: Factors associated with late HCV diagnosis among people with HCV notification and ESLD diagnosis

Univariable and multivariable logistic regression were performed to assess factors associated with late HCV diagnosis. The analysis included people notified with HCV and diagnosed with ESLD during January 1, 2016 - December 31, 2021. Statistical significance was set at p <0.05, with all p values being two-sided. Variables with p ≤0.250 in the unadjusted model, or those with known clinical significance, were considered for the adjusted model.

Analysis 4: Factors associated with late or no treatment among people with HCV notification and ESLD diagnosis

Similar statistical methods were employed to examine factors associated with late or no treatment among those notified with HCV and diagnosed with ESLD during January 1, 2016 - December 31, 2021, defined as either late HCV treatment (occurring within 2 years prior or following ESLD diagnosis) or no HCV treatment. The same statistical approach and significance criteria as described in Analysis 3 were applied. All analyses were performed using Stata v18.0. Visualisation of data was presented using GraphPad Prism 10.2.2 (Insight Partners, NY, USA).

Results

Study cohort

The study cohort included 4,419 people with an HCV notification made in NSW between January 1, 1995, to March 31, 2022, and diagnosed with ESLD between January 1, 2010, to December 31, 2021 (Table 1). Over the study period, the cohort had relatively even distribution of ESLD cases: 25% (n = 1,078) in 2010-2012, 26% (n = 1,185) in 2013-2015, 26% (n = 1,160) in 2016-2018, and 23% (n = 993) in 2019-2021. The cohort was predominantly male (75%), Australian-born (83%), and born between 1945-1959 (45%) or 1960-1974 (41%). Most participants resided in rural/regional (38%) or outer metropolitan (35%) areas, with fewer living in metropolitan regions (24%). Among the cohort, 51% were categorised with recent injecting drug use, and 51% had recent alcohol use disorder.

Late HCV diagnosis

During 2010-2021, late HCV diagnoses decreased over time: from 24% of ESLD cases in 2010-2012 to 16% in 2019-2021 (p <0.001) (Fig. 1, Table 2). Across all periods, late diagnosis was more common among males, 24% in 2010-2012, declining to 18% in 2019-2021. Among females, late diagnosis was 22% in 2010-2012, declining to 12% in 2019-2021. Late diagnosis was more common among people born in 1975 or later, 41% in 2010-2012, declining to 20% in 2019-2021. Late diagnosis was more common among people residing in rural/regional areas, 29% in 2010-2012, declining to 18% in 2019-2021. Among people with recent alcohol use disorder, late diagnosis was 29% in 2010-2012, declining to 18% in 2019-2021. Among people with recent incarceration history, late diagnosis was 32% in 2010-2012 and declined to 10% in 2019-2021.

The proportion of late HCV diagnoses among people with DC decreased during the study period from 25% (n = 166/677) in 2010-2012 to 17% (n = 94/540) in 2019-2021 (p <0.001) (Fig. S1). The proportion of late HCV diagnoses among people with HCC decreased from 20% (n = 68/337) in 2010-2012 to 14% (n = 72/530) in 2019-2021 (p <0.001).

Timing of HCV treatment

Among people notified with HCV and diagnosed with ESLD in 2010-2012, 2% (n = 18/1,081) of people received early treatment, 13% (n = 148/1,081) received late treatment, and most

Table 1. Demographic characteristics among NSW people with an HCV notification and ESLD, 2010-2021.

	2010-2021		2010-2012		2013-2015		2016-2018		2019-2021	
**Characteristics, n (%)**	**n (col%)**		**n (col%)**		**n (col%)**		**n (col%)**		**n (col%)**	
Total, n (row%)		4,419	1,081	25	1,185	26	1,160	26	993	23
Birth cohort										
≤1944	362	8	134	12	105	9	69	6	54	5
1945-1959	1,982	45	533	49	536	45	511	44	388	39
1960-1974	1,807	41	365	34	482	41	488	42	472	48
≥1975	282	6	49	5	62	5	92	8	79	8
Sex[a]										
Male	3,331	75	811	75	900	76	889	77	731	74
Female	1,080	24	268	25	283	24	270	23	259	26
Region of HCV notification[a]										
Metropolitan	1,083	24	290	27	303	26	264	23	226	23
Outer metropolitan	1,542	35	377	35	428	36	410	35	327	33
Rural/Regional	1,692	38	392	36	435	37	459	40	406	41
Country of birth[a]										
Australia	3,652	83	865	80	987	83	964	83	836	84
Overseas	767	17	216	20	198	17	196	17	157	16
Injecting drug use										
No history	1,545	35	363	34	392	33	403	35	387	39
Distant	621	14	155	14	168	14	163	14	135	14
Recent	2,253	51	563	52	625	53	594	51	471	47
Opioid agonist therapy										
No history	3,388	77	861	79	917	77	868	75	745	75
Distant	793	15	136	13	181	15	195	17	166	17
Recent	350	8	84	8	87	7	97	8	82	8
Alcohol use disorder										
No history	1,839	42	460	43	452	38	480	41	447	45
Distant	342	7	89	8	77	7	95	8	81	8
Recent	2,238	51	532	49	656	55	585	50	465	47
Incarceration										
No history	3,388	77	885	82	929	78	870	75	701	71
Distant	793	18	142	13	209	18	211	18	231	23
Recent	238	5	54	5	47	4	79	7	58	6
Government assistance										
No	418	9	114	11	110	9	111	10	83	8
Distant	554	13	131	12	145	12	148	12	130	13
Recent	3,447	78	836	77	930	79	901	78	780	79

[a]Among people with available information. HCV, hepatitis C virus; NSW, New South Wales.

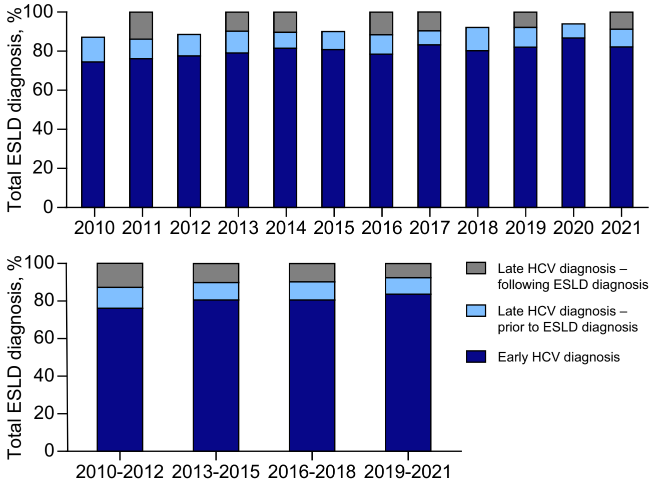

Fig. 1. Proportion of HCV diagnosis timeliness among people with an ESLD diagnosis, 2010-2021. People with an HCV notification and ESLD diagnosis (n = 4,419). Late HCV diagnosis – following (within a month or after ESLD diagnosis); late HCV diagnosis – prior (0-2 years prior to ESLD diagnosis); early HCV diagnosis (>2 years prior to ESLD diagnosis). Levels of significance: p <0.001 (Chi-squared test). ESLD, end-stage liver disease.

were untreated (85%, n = 915/1,081) (Fig. 2). In 2019-2021, 30% (n = 298/993) received early treatment, 22% (n = 216/993) received late treatment, and 48% (n = 479/993) were untreated (Fig. 2, Table S4). Thus, the proportion with a missed treatment opportunity to prevent ESLD (no or late treatment) decreased from 98% to 70% over the study period.

Missed treatment opportunity (no or late treatment) during the DAA era (2016-2018 and 2019-2021 periods) was further characterised. No or late treatment was more common among males, 93% in 2016-2018 and 70% in 2019-2021, compared to females, 88% in 2016-2018 and 69% in 2019-2021 (Table S5). No or late treatment was also common among people born between 1960-1974: 94% in 2016-2018 declining to 72% in 2019-2021. No or late treatment was also common among people with recent history of alcohol use disorder: 95% in 2016-2018 declining to 76% by 2019-2021. Similarly, no or late treatment was common among people with recent OAT (95% in 2016-2018 declining to 71% in 2019-2021), among people with recent injecting drug use (94% to 70%, respectively), and among those with recent incarceration history (94% to 66%, respectively).

Among people notified with HCV and DC, between 2010-2012 and 2019-2021, people who received early treatment

Table 2. **Demographic characteristics among NSW people with an HCV notification and ESLD, by timeliness of HCV diagnosis, 2010-2021.**

	2010-2012				2013-2015				2016-2018				2019-2021			
**Characteristics, n (row%)**	**Late diagnosis, n**	**%**	**Early diagnosis, n**	**%**	**Late diagnosis, n**	**%**	**Early diagnosis, n**	**%**	**Late diagnosis, n**	**%**	**Early diagnosis, n**	**%**	**Late diagnosis, n**	**%**	**Early diagnosis, n**	**%**
Total, n (row%)	258	24	823	76	231	20	954	80	226	19	934	81	163	16	830	84
Birth cohort																
≤1944	22	16	112	84	16	15	89	85	6	9	63	91	8	15	46	85
1945-1959	126	24	407	76	109	20	427	80	101	20	410	80	65	17	323	83
1960-1974	90	25	275	75	84	17	398	83	98	20	390	80	74	16	398	84
≥1975	20	41	29	59	22	36	40	64	21	23	71	77	16	20	63	80
Sex[a]																
Male	198	24	613	76	183	20	717	80	179	20	710	80	131	18	600	82
Female	60	22	208	78	48	17	235	83	47	17	223	83	31	12	228	88
Region of HCV notification[a]																
Metropolitan	53	15	237	85	60	20	243	80	42	16	222	84	32	14	194	86
Outer metropolitan	80	21	297	79	70	16	358	84	68	17	342	83	51	16	276	84
Rural/Regional	113	29	279	71	95	22	340	78	105	23	354	77	72	18	334	82
Country of birth[a]																
Australia	213	25	652	75	192	20	795	80	188	20	776	80	133	16	703	84
Overseas	45	21	171	79	39	20	159	80	38	19	158	81	30	19	127	81
Injecting drug use																
No history	95	27	268	73	81	21	311	79	94	23	309	77	94	42	293	58
Distant	33	21	122	79	26	16	142	84	25	15	138	85	6	4	129	96
Recent	130	23	433	77	124	20	501	80	107	18	487	82	63	13	408	87
Opioid agonist therapy																
No history	228	27	633	73	199	22	718	78	188	22	672	78	147	20	598	80
Distant	22	16	114	84	19	11	162	89	25	13	179	87	8	5	158	95
Recent	8	10	76	90	13	15	74	85	13	13	83	87	8	10	74	90
Alcohol use disorder																
No history	96	21	364	79	82	18	370	82	88	18	392	82	71	16	376	84
Distant	10	11	79	89	6	8	71	92	13	14	82	86	8	10	73	90
Recent	152	29	380	71	143	22	513	78	125	27	460	77	84	18	381	82
Incarceration																
No history	213	24	672	76	189	20	740	80	186	21	684	79	135	19	569	81
Distant	28	20	114	80	31	15	178	85	27	13	184	87	22	10	209	90
Recent	17	32	37	68	11	23	36	77	13	17	66	83	6	10	52	90
Government assistance																
No	33	29	81	71	29	26	81	74	34	31	77	69	20	24	63	76
Distant	46	35	85	65	32	22	113	78	37	25	111	75	29	22	101	78
Recent	179	21	657	79	170	18	760	82	155	17	746	83	114	15	666	85

[a]Among people with available information. HCV, hepatitis C virus; NSW, New South Wales.

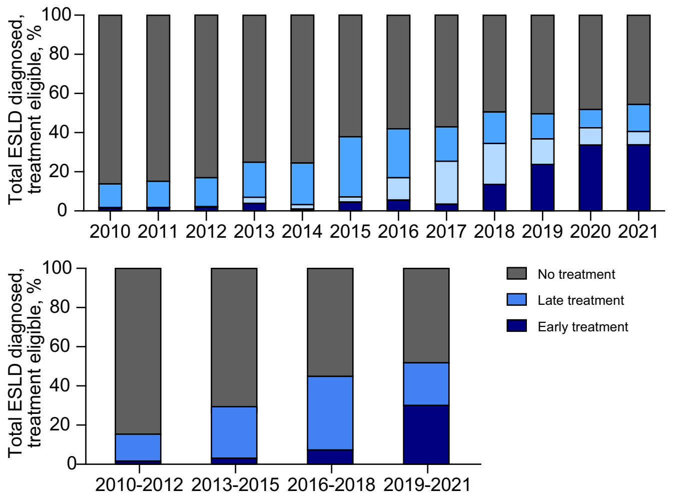

Fig. 2. Proportion of HCV treatment timeliness among people with HCV and an ESLD diagnosis, 2010-2021. People with an HCV notification and ESLD diagnosis (n = 4,419). Late treatment (after or within 2 years prior to ESLD diagnosis); early treatment (>2 years prior to ESLD diagnosis). Levels of significance: *p* <0.001 (Chi-squared test). ESLD, end-stage liver disease.

increased from 1% (7/677) to 25% (131/540) (*p* <0.001), late HCV treatment increased from 15% (99/677) to 24% (n = 129/540) (*p* <0.001), and no treatment decreased from 84% (n = 571/677) to 52% (n = 280/540) (*p* <0.001) (Fig. 3). Among people notified with HCV and HCC between 2010–2012 and 2019–2021, the proportion who received early treatment increased from 2% (9/337) to 40% (214/530) (*p* <0.001), the proportion receiving late treatment increased from 8% (26/337) to 18% (95/530) (*p* <0.001), and the proportion who remained untreated decreased from 90% (302/337) to 42% (221/530) (*p* <0.001) (Fig. 3).

Factors associated with late HCV diagnosis among people with HCV notification and ESLD diagnosis in NSW, 2016-2021

Individuals residing in rural/regional areas (adjusted odds ratio [aOR] 1.44, 95% CI 1.05-1.97, *p* = 0.024) had higher odds of late HCV diagnosis. In contrast, several variables related to social marginalisation were associated with lower odds of late HCV diagnosis. Injecting drug use was associated with lower odds of late HCV diagnosis (adjusted OR [aOR] 0.43, 95% CI 0.28-0.65, *p* = 0.001 for distant use; aOR 0.78, 95% CI 0.60-0.99, *p* = 0.041 for recent use, compared to no injecting drug use) (Table 3). Incarceration history was associated with lower odds of late HCV diagnosis, with distant incarceration (aOR 0.55, 95% CI 0.38–0.78, *p* = 0.001) and recent incarceration (aOR 0.51, 95% CI 0.28–0.96, *p* = 0.037) having reduced odds compared to no incarceration history. Recent receipt of government assistance was also associated with reduced odds of late HCV diagnosis (aOR 0.57, 95% CI 0.39-0.82, *p* = 0.002). There were lower odds of late diagnosis among those born on or before 1944 (aOR 0.31, 95% CI 0.15-0.66, *p* = 0.002) and those born between 1945-1959 (aOR 0.47, 95 CI% 0.29-0.77, *p* = 0.003), compared to those born on or after 1975.

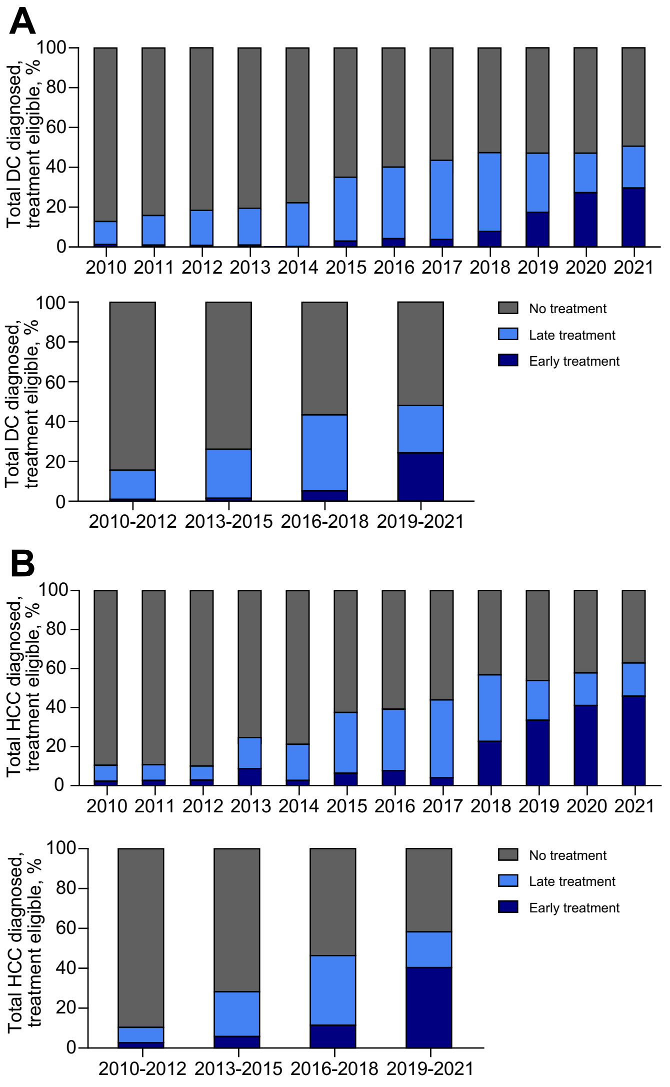

Fig. 3. Trends in HCV treatment timeliness among people with HCV and a DC or HCC diagnosis, 2010-2021. People with an HCV notification and ESLD diagnosis (n = 4,419). (A) People with a DC diagnosis (n = 2,580). (B) People with a HCC diagnosis (n = 1,839). Late treatment (after or 2 years prior to ESLD diagnosis; early treatment (>2 years prior to ESLD diagnosis). Levels of significance: *p* <0.001 (Chi-squared test). DC, decompensated cirrhosis; ESLD, end-stage liver disease; HCC, hepatocellular carcinoma.

Factors associated with timing of HCV treatment among people with HCV notification and ESLD diagnosis, 2016-2021

In the adjusted model, recent alcohol use disorder was associated with no or late HCV treatment (aOR 1.80, 95% CI 1.40-2.32, *p* = 0.001) (Table 4). Those born between 1945-1959 (aOR 0.58, 95% CI 0.34-0.98, *p* = 0.041) was associated with lower odds of no or late HCV treatment compared to those born on or after 1975.

Discussion

The WHO global strategy to eliminate viral hepatitis as a public health threat emphasises early diagnosis and timely treatment of HCV to prevent progression to ESLD.[13] Our study leveraging

Table 3. Factors associated with late HCV diagnosis among people with HCV notification and an ESLD diagnosis in NSW, 2016-2021.

Characteristic	OR (95 % CI)	*p* value*	Adjusted OR (95% CI)	*p* valueˉ
Birth cohort				
≤1944	0.47 (0.24-0.90)	0.024	0.31 (0.15-0.66)	0.002
1945-1959	0.82 (0.55-1.22)	0.333	0.47 (0.29-0.77)	0.003
1960-1974	0.79 (0.53-1.18)	0.249	0.64 (0.40-1.02)	0.060
≥1975	reference		reference	
Sex				
Male	reference		reference	
Female	1.37 (1.04-1.79)	0.023	1.29 (0.97-1.71)	0.085
Region of HCV notification				
Metropolitan	reference		reference	
Outer metropolitan	1.08 (0.79-1.49)	0.623	1.11 (0.97-1.71)	0.530
Rural/Regional	1.45 (1.07-1.95)	0.015	1.44 (1.05-1.97)	0.024
Country of birth				
Australia	reference		reference	
Overseas	1.10 (0.82-1.47)	0.523	1.13 (0.81-1.58)	0.472
Injecting drug use				
No	reference		reference	
Distant	0.37 (0.25-0.56)	0.001	0.43 (0.28-0.65)	0.001
Recent	0.61 (0.48-0.77)	0.001	0.78 (0.60-0.99)	0.041
Opioid agonist therapy				
No	reference		reference	
Distant	0.26 (0.17-0.41)	0.001	0.32 (0.20-0.51)	0.001
Recent	0.52 (0.33-0.82)	0.005	0.59 (0.35-1.00)	0.052
Alcohol use disorder				
No	reference		reference	
Distant	0.66 (0.40-1.07)	0.088	0.78 (0.47-1.29)	0.336
Recent	1.20 (0.96-1.51)	0.117	1.24 (0.96-1.61)	0.098
Incarceration				
No	reference		reference	
Distant	0.9 (0.35-0.67)	0.001	0.55 (0.38-0.78)	0.001
Recent	0.63 (0.38-1.04)	0.069	0.51 (0.28-0.96)	0.037
Government assistance				
No	reference		reference	
Distant	0.81 (0.53-1.23)	0.315	0.82 (0.53-1.26)	0.336
Recent	0.49 (0.35-0.69)	0.001	0.57 (0.39-0.82)	0.002

ESLD, end-stage liver disease; NSW, New South Wales; OR, odds ratio.

ORs and 95% CIs were estimated using univariable and multivariable logistic regression models. *Statistical significance for the univariable logistic regression model. ˉStatistical significance for the multivariable logistic regression model.

NSW population-level data, demonstrates continued improvements in timeliness of HCV care among people diagnosed with ESLD**,** who represent the clinical sequelae of delayed intervention. Over the past decade, declining late HCV diagnoses and increasing uptake and earlier initiation of treatment have been observed. Improved timeliness of HCV diagnosis has been a steady trend over the 12-year study period, whereas timely treatment has been largely evident in the DAA era, where government-subsidised antiviral therapy and Australia's public health response have transformed HCV care and outcomes for all priority populations.

The decline in late HCV diagnosis (24% in 2010-2012 to 16% in 2019-2021), is encouraging and reflects continued improvements in HCV screening and integrated care initiatives. The NSW Government Hepatitis C Strategy 2022-2025 promotes embedding HCV testing in key settings where priority populations interact.[14] Harm reduction strategies including screening within correctional facilities and integration of care within OAT programs, have proven instrumental in diagnosing HCV earlier among people with histories of injecting drug use and incarceration.[15,16] The impact of these initiatives is underscored by our study, which found that people with recent injecting drug use or recent incarceration were less likely to have a late HCV diagnosis. This highlights the success of targeted efforts in NSW to engage at-risk groups, although further reductions in late HCV diagnoses will require ongoing efforts to address barriers to HCV testing. The recent implementation of point-of-care technology has enhanced testing capabilities, enabling quicker and more accessible diagnosis, particularly in high-risk settings,[17] which should further improve timeliness of HCV diagnosis.

Our findings align and build upon our initial work evaluating late HCV diagnosis in NSW during 2001-2012, which provided the foundation for examining this issue in the Australian context.[7] Our early work introduced an operational definition of late HCV diagnosis based on a 2-year period prior to or following first hospitalisation for ESLD, a pragmatic approach based on available population-level linkage data ensuring real-world applicability through administrative healthcare data. This definition has been utilised by other Australian[18] and international groups,[8] while alternative definitions that utilise liver fibrosis staging at presentation have also been used.[6] Collectively, these studies have reported declines in late HCV diagnosis over time, but several also highlight major missed opportunities, even among individuals with multiple interactions with the healthcare system. These persistent gaps in timely diagnosis highlight the need for more comprehensive surveillance and intervention strategies. Our current analysis extends these findings by providing contemporary data through 2021 and specifically examining the intersection of both late HCV

Table 4. Factors associated with missed treatment opportunity[a] among people with HCV notification and ESLD diagnosis in NSW, 2016-2021.

Characteristic	OR (95 % CI)	*p* value*	Adjusted OR (95% CI)	*p* value ˆ
Birth cohort				
≤1944	1.27 (0.63-2.57)	0.504	1.31 (0.60-2.88)	0.491
1945-1959	0.64 (0.40-1.01)	0.057	0.58 (0.34-0.98)	0.041
1960-1974	0.80 (0.51-1.28)	0.357	0.69 (0.42-1.15)	0.155
≥1975	reference		reference	
Sex				
Male	reference		reference	
Female	1.08 (0.84-1.39)	0.537	1.13 (0.86-1.47)	0.383
Region of HCV notification				
Metropolitan	reference		reference	
Outer metropolitan	0.98 (0.73-1.32)	0.895	0.97 (0.72-1.32)	0.857
Rural/Regional	0.96 (0.72-1.29)	0.791	0.96 (0.71-1.29)	0.783
Country of birth				
Australia	reference		reference	
Overseas	1.04 (0.77-1.41)	0.785	1.08 (0.78-1.52)	0.639
Injecting drug use				
No	reference		reference	
Distant	1.06 (0.75-1.49)	0.733	1.04 (0.73-1.48)	0.812
Recent	1.22 (0.96-1.55)	0.102	1.11 (0.85-1.45)	0.435
Opioid agonist therapy				
No	reference		reference	
Distant	0.94 (0.70-1.26)	0.682	0.94 (0.68-1.31)	0.721
Recent	1.28 (0.83-1.99)	0.259	1.25 (0.76-204)	0.380
Alcohol use disorder				
No	reference		reference	
Distant	0.86 (0.59-1.26)	0.441	0.92 (0.62-1.36)	0.672
Recent	1.70 (1.34-2.15)	0.001	1.80 (1.40-2.32)	0.001
Incarceration				
No	reference		reference	
Distant	0.99 (0.76-1.31)	0.967	0.84 (0.62-1.17)	0.325
Recent	1.02 (0.64-1.61)	0.937	0.85 (0.49-1.44)	0.538
Government assistance				
No	reference		reference	
Distant	0.79 (0.49-1.28)	0.358	0.78 (0.48-1.26)	0.310
Recent	0.97 (0.65-1.44)	0.952	0.88 (0.58-1.32)	0.531

ESLD, end-stage liver disease; NSW, New South Wales; OR, odds ratio.
ORs and 95% CIs were estimated using univariable and multivariable logistic regression models. *Statistical significance for the univariable logistic regression model. ˆStatistical significance for the multivariable logistic regression model.
Were suppressed in this analysis.
[a]Missed treatment opportunity includes people with late and no HCV treatment.

diagnosis and treatment with ESLD outcomes, offering valuable insights into the long-term impact of delayed HCV intervention.

Timely therapeutic intervention for HCV, particularly with highly curative DAA regimens, reduces the risk of DC and HCC.[3] Evaluation of the timeliness of HCV treatment among those with ESLD should be approached with caution, as individuals who progress to advanced liver disease represent a selected population more likely to have missed opportunities for earlier therapeutic intervention. However, monitoring of timeliness of HCV treatment in this population can be informative. The marked reduction in no or late HCV treatment that we have defined as "missed treatment opportunity" (98% in 2010-2012 to 70% in 2019-2021), alongside an increase in HCV treatment initiation (15% in 2010-2012 to 52% in 2019-2021), reflects the transformative impact of Australia's unrestricted DAA program since 2016. Targeted interventions and comprehensive care models, including those in marginalised communities, have played a pivotal role in achieving high HCV treatment coverage.[15] The initial rapid DAA scale up and its relative equity – no social marginalisation factors that were analysed in our study were associated with missed treatment opportunity – are hallmarks of Australia's successful HCV response. But, given declining HCV treatment delivery and our finding that a majority of those with ESLD have still missed the opportunity of earlier intervention, it is not surprising that modelling suggests renewed efforts are required to achieve elimination targets.[19]

While early treatment for people diagnosed with HCC increased from 2% in 2010-2012 to 40% in 2019-2021, somewhat surprisingly the increase was less for people with a DC diagnosis during the same period (1% to 25%). Part of the explanation may relate to the compassionate DAA access program, a mechanism that began in late 2014 prior to Australian Government funding, prioritising people with cirrhosis, but excluding those with HCC.[20] An estimated 4,385 people were treated through this program between late 2014 and early 2016.[21] The increase in early treatment for patients with HCC likely suggests a combination of broader shifts in clinical practice and healthcare engagement in Australia. Integration of HCV treatment into multidisciplinary HCC management and evolving clinical guidelines placed greater emphasis on antiviral therapy post-curative treatment, further reinforcing DAAs' role in HCC management.[22] Despite initial exclusion from early access programs, patients with HCC ultimately demonstrated greater treatment uptake once universal access became available. In contrast, the lower increase in early treatment for patients with DC may seem counterintuitive given

the initial prioritisation of those with cirrhosis in compassionate access programs, but likely reflects persistent barriers to timely intervention including the more limited DAA therapy options for patients with advanced liver disease.[23]

The NSW HCV response has enhanced timeliness of HCV diagnosis and treatment, but its impact has differed across geographic regions. People residing in rural and regional areas were more likely to have delayed HCV diagnosis, reflecting an ongoing challenge faced by rural Australians accessing healthcare. Research examining DAA treatment completion in Australia during the study period likewise observed lower completion rates in remote areas with decreasing uptake.[24] Despite a higher per capita spend in healthcare with increasing remoteness in Australia, this does not result in additional activity for a myriad of reasons, including housing, food security and associated costs.[25] The competing priorities between essential living needs and accessing healthcare often delay access to care. The remoteness of those living in rural Australia also creates physical barriers to accessing health services, requiring a greater time commitment to reach them.[26] The resulting impact is concerning, characterised by increased disease severity at diagnosis, delayed presentation, and higher prevalence.[25,27] The impact is likely to disproportionately affect Aboriginal and Torres Strait Islander communities, who are overrepresented in rural and remote areas and face compounded challenges of culturally unsafe healthcare services, and historical mistrust of health systems.[28] Aboriginal Community Controlled Health Services are limited in availability in certain rural areas – addressing these barriers requires continued investment in culturally safe care models and expanded outreach efforts in Aboriginal communities.

Recent developments in remote health delivery, several catalysed by the COVID-19 pandemic, align with the NSW strategy and may further alleviate these barriers. Strategies include telehealth services,[29] remote prescribing capabilities,[30] decentralising models of care,[31] and nurse-led models of care.[32] Mobile health clinics and community-led outreach programs in Australia have also demonstrated success in increasing engagement in HCV care by providing decentralised rapid testing and treatment services directly to rural populations.[33] Expanding these initiatives, alongside further investment in primary care-HCV integration, could help bridge access gaps. Strategic targeting of preventative health programs has also been implemented to enhance pathways to HCV testing and treatment initiation,[17] and may further reduce healthcare access disparities in rural and regional communities.

By contrast, people who recently received government assistance were less likely to experience late HCV diagnosis, suggesting a critical role of these resources in facilitating access to HCV care. Studies demonstrate access to support services and implementation of financial incentives can reduce barriers to care and promote timely detection and treatment, emphasising the importance of patient-centred care models.[17]

Social marginalisation factors such as recent injecting drug use and recent incarceration were associated with earlier HCV diagnosis and had no association with missed HCV treatment opportunity, a testament to the relative equity of HCV services in NSW. In contrast, alcohol use disorder was associated with a higher risk of missed treatment opportunity. This finding raises several concerns, as those with alcohol use disorder and HCV have an increased risk of advanced liver disease complications,[34] and therefore would potentially benefit more from earlier therapeutic intervention for HCV. Despite efforts to integrate HCV care within healthcare and community settings, including alcohol and drug services in NSW,[15] barriers to treatment initiation remain. These may include healthcare provider biases, structural obstacles, and stigma,[35] often stemming from provider concerns over adherence among patients with active alcohol use.[36] While some evidence suggests lower adherence among those with heavy drinking,[37] studies also show relatively high rates of treatment success even among patients with excessive alcohol intake.[38] This aligns with many international HCV treatment guidelines that increasingly do not exclude patients based solely on active or unhealthy alcohol consumption.[39] However, concerns over adherence and the likelihood of treatment initiation are not limited to alcohol use; concurrent health issues, such as active drug use or mental health conditions, can also lead providers to deprioritise and subsequently delay HCV treatment.[36] While addressing these barriers requires targeted interventions, and proactive case management to support engagement and adherence,[40] it also demands provider education to improve HCV care delivery, particularly for patients with complex health concerns, as trust in the provider-patient relationship is vital.[35]

Embedding HCV care in alcohol, drug, and mental health services not only alleviates barriers but has the potential to enhance treatment adherence and improve long-term outcomes for patients and should be expanded and promoted. Such models could improve treatment uptake by addressing the complex interplay between concurrent conditions, offering holistic care that supports patients' overall well-being and treatment adherence.[41]

Drawing lessons from successful implementations elsewhere can further inform NSW's approach. Beyond local integrated care models, endeavours across the US and Europe, such as HCV care delivered from shelters[42] and through mobile street outreach,[43] offer innovative approaches that enhance healthcare accessibility for marginalised populations. Several low- and middle-income countries have adopted HCV care task-shifting strategies[44] and implemented decentralised hub-and-spoke models for point-of-care testing[45] to improve access despite resource constraints. These global models demonstrate how integrated care can tackle health inequities while also addressing broader healthcare system challenges, underscoring the importance of overcoming barriers to HCV care.

Beyond improving health outcomes, addressing patient, provider, and system barriers to HCV care is crucial but also has significant economic implications. Early intervention for HCV provides significant benefits for both patients and the healthcare system by reducing the burden of advanced liver disease.[46] In NSW, the expansion of HCV treatment between 2016 and 2022 resulted in estimated healthcare cost savings of AUD$103 million.[47] Furthermore, liver-related mortality declined by 15% during this period, following a period of escalating mortality, demonstrating the positive impact of antiviral treatments and care initiatives in NSW.[47] These positive outcomes suggest even greater cost benefits could be achieved through earlier intervention, particularly given that the total associated costs for HCC in Australia in 2019-2020 were estimated at AUD$4.8 billion.[48]

There are several limitations to this study that should be acknowledged, and findings should be interpreted accordingly.

First, the study population is limited to people who engaged with the healthcare system and received an ESLD diagnosis at hospitalisation. By evaluating late diagnosis and treatment in the context of ESLD, the study selected the most severe cases of chronic HCV infection, generally those infected for at least two decades. Second, the analysis excludes individuals with advanced liver disease who were managed solely as outpatients or remained undiagnosed. This may underestimate the true burden of late HCV diagnosis in NSW. However, earlier work examining hospitalisation records against the cancer registry demonstrated high concordance for HCC diagnosis, suggesting that our approach captured the majority of clinically significant cases.[49] Third, our analysis likely underestimates the impact of several important factors: healthcare accessibility differences between urban and rural populations, and the experiences of marginalised individuals with limited system engagement. While we identified individuals with social marginalisation through healthcare and social service records, this approach likely misses those with severe social exclusion who have minimal formal system contact. Contextual influences such as stigma, health literacy, and competing life priorities further shape HCV care patterns in ways our study cannot fully capture. As a result, our findings may reflect only the most visible portion of late HCV diagnosis, with the actual prevalence likely being higher in the broader population. Fourth, while the study period (2019–2021) overlaps with the COVID-19 pandemic, we did not observe a sharp decline in HCV testing, treatment initiation, or ESLD-related hospitalisations. However, delays in HCV care may still emerge over time[50] and continued surveillance is needed to assess potential lagging effects beyond 2021. Lastly, although the study focuses exclusively on NSW, the findings may not fully generalise to other regions or countries with different healthcare infrastructures. Future research should aim to address these gaps to provide a more comprehensive view of HCV care outcomes.

In conclusion, this study provides evidence of declining late HCV diagnoses and missed treatment opportunities, demonstrating the effectiveness of unrestricted DAAs and NSW's elimination strategy in addressing the unique needs of marginalised populations. It also underscores the importance of strengthening these targeted approaches as NSW progresses toward its elimination goals. Future strategies should focus on interventions that address delayed diagnosis and missed treatment opportunities, including embedding HCV care in drug and alcohol services and linking rural Australians to care to ensure broader access and equitable treatment outcomes.

Affiliations

[1]*The Kirby Institute, UNSW Sydney, Sydney, NSW, Australia;* [2]*Storr Liver Centre, The Westmead Institute for Medical Research, Westmead Hospital, NSW, Australia;* [3]*University of Sydney, Sydney, NSW, Australia;* [4]*Department of Health Sciences, Faculty of Medicine and Health Sciences, Macquarie University, Sydney, NSW, Australia;* [5]*School of Population Health, UNSW Sydney, Sydney, Australia*

Abbreviations

aOR, adjusted odds ratio; DAA, direct-acting antiviral; DC, decompensated cirrhosis; ESLD, end-stage liver disease; HCC, hepatocellular carcinoma; OAT, opioid agonist therapy.

Conflict of interest

GD reports research support from Gilead and Abbvie. HV has received honoraria from Gilead Sciences. JGe received consulting fees from NovoNordisk, AbbVie, Gilead Sciences, BMS, Pharmaxis, Novartis, Cincera, Pfizer, Roche, Eisai and Bayer. JGr has received research grants from AbbVie, Biolytical, Cepheid, Gilead and Hologic, and has received honoraria from AbbVie, Abbott, Cepheid, Gilead and Roche outside the submitted work. MM has received consulting fees from Abbvie. KP reports grants from Gilead Sciences and ViiV health care. GM reports grants from ViiV and Janssen, received honororia from ViiV and Gilead and participated on a Data Safety Monitoring Board for ViiV. All remaining authors have no potential conflicts to declare. Disclaimer: All inferences, opinions, and conclusions drawn in this publication are those of the author(s), and do not necessarily reflect the opinions or policies of the Australian Government Department of Health.

Please refer to the accompanying ICMJE disclosure forms for further details.

Authors' contributions

Shane Tillakeratne: Writing – review & editing, Writing – original draft, Visualisation, Project administration, Investigation, Formal analysis. Heather Valerio: Writing – review & editing, Supervision, Methodology, Investigation, Conceptualization. Maryam Alavi: Writing –review & editing. Behzad Hajarizadeh: Writing – review & editing. Marianne Martinello: Writing – review & editing. Jacob George: Writing – review & editing. Janaki Amin: Writing – review & editing. Kathy Petoumenos – review & editing. Gail Matthews: Writing – review & editing. Jason Grebely: Writing – review & editing. Sallie-Anne Pearson: Writing – review & editing, Supervision, Methodology. Gregory J. Dore: Writing – review & editing, Supervision, Methodology, Conceptualisation.

Data availability statement

This publication involved information collected by population-based health administration registries. Data used for this research cannot be deposited on servers other than those approved by ethics committees. This publication has used highly sensitive health information through linkage of several administrative datasets. De-identified linked information has been provided to the research team under strict privacy regulations. Except in the form of conclusions drawn from the data, researchers do not have permission to disclose any data to any person other than those authorised for the research project.

Ethics approval

This study was approved by NSW Population and Health Services Research Ethics Committee (Approval number: 2019/ETH01777) and the Australian Institute of Health and Welfare Ethics Committee (Approval number: EO2021/3/1274).

Financial support

The Kirby Institute, UNSW Sydney, and New South Wales Ministry of Health, Australia. The Kirby Institute is funded by the Australian Government Department of Health, under the agreement ID number 2-D3X513. This publication is part of the Bloodborne viruses and sexually transmissible infections Research, Strategic Interventions and Evaluation programme, funded by the New South Wales Ministry of Health. JGr is supported by an Australian National Health and Medical Research Council Investigator Grant (1176131). MM is supported by an Australian National Health and Medical Research Council Investigator Grant (2034418). Gregory J. Dore is supported through a National Health and Medical Research Council Investigator Grant (2008276).

Acknowledgements

We would like to acknowledge the New South Wales Ministry of Health Centre for Health Record Linkage for the provision of HCV notifications, hospital admissions, incarcerations, OAT authority, and death data. We would like to thank the ethics committees of New South Wales Population and Health Services Research, New South Wales Ministry of Health, for their approval of this publication. We would like to thank Jessica Respecio for her expertise with the formatting of the graphical abstract for this manuscript.

Supplementary data

Supplementary data to this article can be found online at https://doi.org/10.1016/j.jhepr.2025.101474.

References

Author names in bold designate shared co-first authorship.

[1] Martinello M, Hajarizadeh B, Grebely J, et al. Management of acute HCV infection in the era of direct-acting antiviral therapy. Nat Rev Gastroenterol Hepatol 2018;15(7):412–424.

[2] World Health Organization. Interim guidance for country validation of viral hepatitis elimination, 2021. Available from: https://www.who.int/publications/i/item/9789240028395.

[3] Nahon P, Bourcier V, Layese R, et al. Eradication of hepatitis C virus infection in patients with cirrhosis reduces risk of liver and non-liver complications. Gastroenterology 2017;152(1). 142-56.e2.

[4] McDonald SA, Barclay ST, Innes HA, et al. Uptake of interferon-free DAA therapy among HCV-infected decompensated cirrhosis patients and evidence for decreased mortality. J Viral Hepat 2021;28(9):1246–1255.

[5] Tillakeratne S, Pearson S-A, Alavi M, et al. Trends in viral hepatitis liver-related morbidity and mortality in New South Wales, Australia. Lancet Reg Health – West Pac 2024;51.

[6] Moorman AC, Xing J, Ko S, et al. Late diagnosis of hepatitis C virus infection in the Chronic Hepatitis Cohort Study (CHeCS): missed opportunities for intervention. Hepatology 2015;61(5):1479–1484.

[7] Alavi M, Law MG, Grebely J, et al. Time to decompensated cirrhosis and hepatocellular carcinoma after an HBV or HCV notification: a population-based study. J Hepatol 2016;65(5):879–887.

[8] Samji H, Yu A, Kuo M, et al. Late hepatitis B and C diagnosis in relation to disease decompensation and hepatocellular carcinoma development. J Hepatol 2017;67(5):909–917.

[9] Kirby Institute. Hepatitis B and C in Australia annual surveillance report supplement 2016. Australia: Kirby Institute, UNSW Sydney; 2016.

[10] NSW Ministry of Health. Improved reporting of aboriginal and Torres Strait Islander peoples on population datasets in New South Wales using record linkage–A feasibility study. 2012. Sydney, NSW, Australia.

[11] Valerio H, Alavi M, Law M, et al. High hepatitis C treatment uptake among people with recent drug dependence in New South Wales, Australia. J Hepatol 2021;74(2):293–302.

[12] Friedmann PD. Alcohol use in adults. New Engl J Med 2013;368(4):365–373.

[13] World Health Organization. Global health sector strategy on viral hepatitis 2016–2021. 2016 [Available from: https://www.who.int/hepatitis/strategy2016-2021/ghss-hep/en/.

[14] NSW Heath. NSW hepatitis C strategy 2022 - 2025. 2022.

[15] Valerio H, Alavi M, Conway A, et al. Declining prevalence of current HCV infection and increased treatment uptake among people who inject drugs: the ETHOS Engage study. Int J Drug Policy 2022;105:103706.

[16] Hajarizadeh B, Grebely J, Byrne M, et al. Evaluation of hepatitis C treatment-as-prevention within Australian prisons (SToP-C): a prospective cohort study. Lancet Gastroenterol Hepatol 2021;6(7):533–546.

[17] Cunningham EB, Wheeler A, Hajarizadeh B, et al. Interventions to enhance testing, linkage to care, and treatment initiation for hepatitis C virus infection: a systematic review and meta-analysis. Lancet Gastroenterol Hepatol 2022;7(5):426–445.

[18] Mnatzaganian G, MacLachlan JH, Allard N, et al. Missed opportunities for diagnosis of hepatitis B and C in individuals diagnosed with decompensated cirrhosis or hepatocellular carcinoma. J Gastroenterol Hepatol 2023;38(6):976–983.

[19] Kwon JA, Dore GJ, Hajarizadeh B, et al. Australia could miss the WHO hepatitis C virus elimination targets due to declining treatment uptake and ongoing burden of advanced liver disease complications. PLoS One 2021;16(9):e0257369.

[20] Hajarizadeh B. Monitoring hepatitis C treatment uptake in Australia (Issue 13). Australia: Kirby Institute, UNSW Sydney; 2023.

[21] Kirby Institute. Monitoring hepatitis C treatment uptake in Australia. Australia: Kirby Institute, UNSW Sydney; 2020.

[22] Lubel JS, Roberts SK, Strasser SI, et al. Australian recommendations for the management of hepatocellular carcinoma: a consensus statement. Med J Aust 2021;214(10):475–483.

[23] Gastroenterological Society of Australia. Hepatitis C Virus Infection Consensus Statement Working Group. Australian recommendations for the management of hepatitis C virus infection: a consensus statement. 2002. Melbourne.

[24] Hailstone L, MacLachlan J, Doyle Z, et al. Hepatitis C direct-acting antiviral treatment completion: comparison of geographic, demographic and clinical characteristics in Australia. Aust J Gen Pract 2023;52:391–399.

[25] National Rural Health Alliance. Evidence base for additional investment in rural health in Australia. 2023. Australia.

[26] Barbieri S, Jorm L. Travel times to hospitals in Australia. Scientific Data 2019;6(1):248.

[27] Australian Institute of Health and Welfare. Survey of Health Care: selected findings for rural and remote Australians summary. 2018. Australia.

[28] Nolan-Isles D, Macniven R, Hunter K, et al. Enablers and barriers to accessing healthcare services for Aboriginal people in New South Wales, Australia. Int J Environ Res Public Health 2021;18(6):3014.

[29] Schulz TR, Kanhutu K, Sasadeusz J, et al. Using telehealth to improve access to hepatitis C treatment in the direct-acting antiviral therapy era. J Telemed Telecare 2020;26(3):180–185.

[30] Haridy J, Iyngkaran G, Nicoll A, et al. Outcomes of community-based hepatitis C treatment by general practitioners and nurses in Australia through remote specialist consultation. Intern Med J 2021;51(11):1927–1934.

[31] Lee A, Hanson J, Fox P, et al. A decentralised, multidisciplinary model of care facilitates treatment of hepatitis C in regional Australia. Elsevier; 2018. p. 160–164.

[32] Rodrigues B, Parsons N, Haridy J, et al. A nurse-led, telehealth-driven hepatitis C management initiative in regional Victoria: cascade of care from referral to cure. J Telemed Telecare 2024;30(3):497–504.

[33] Finbråten A-K, Eckhardt BJ, Kapadia SN, et al. Rapid treatment initiation for hepatitis C virus infection: potential benefits, current limitations, and real-world examples. Gastroenterol Hepatol 2022;18(11):628.

[34] Alavi M, Janjua NZ, Chong M, et al. The contribution of alcohol use disorder to decompensated cirrhosis among people with hepatitis C: an international study. J Hepatol 2018;68(3):393–401.

[35] Rogal SS, Arnold RM, Chapko M, et al. The patient-provider relationship is associated with hepatitis C treatment eligibility: a prospective mixed-methods cohort study. PloS one 2016;11(2):e0148596.

[36] Rogal SS, McCarthy R, Reid A, et al. Primary care and hepatology Provider–Perceived barriers to and facilitators of hepatitis C treatment Candidacy and adherence. Dig Dis Sci 2017;62:1933–1943.

[37] Akiyama MJ, Norton BL, Arnsten JH, et al. Intensive models of hepatitis C care for people who inject drugs receiving opioid agonist therapy: a randomized controlled trial. Ann Intern Med 2019;170(9):594–603.

[38] Christensen S, Buggisch P, Mauss S, et al. Alcohol and cannabis consumption does not diminish cure rates in a real-world cohort of chronic hepatitis C virus infected patients on opioid substitution therapy—data from the German Hepatitis C-Registry (DHC-R). Substance Abuse: Res Treat 2019;13:1178221819835847.

[39] Marshall AD, Willing AR, Kairouz A, et al. Direct-acting antiviral therapies for hepatitis C infection: global registration, reimbursement, and restrictions. Lancet Gastroenterol Hepatol 2024;9:366–382.

[40] Boodram B, Kaufmann M, Aronsohn A, et al. Case management and capacity building to enhance hepatitis C treatment uptake at community health centers in a large urban setting. Fam Community Health 2020;43(2):150–160.

[41] Mahoney BJ, Morford KL, Biegacki ET, et al. Hepatitis C virus and integrated care for substance use disorders. Clin Liver Dis 2024;23(1):e0241.

[42] Khalili M, Powell J, Park HH, et al. Shelter-based integrated model is effective in scaling up hepatitis C testing and treatment in persons experiencing homelessness. Hepatol Commun 2022;6(1):50–64.

[43] Trooskin SB, Poceta J, Towey CM, et al. Results from a geographically focused, community-based HCV screening, linkage-to-care and patient navigation program. J Gen Intern Med 2015;30:950–957.

[44] Seidman G, Atun R. Does task shifting yield cost savings and improve efficiency for health systems? A systematic review of evidence from low-income and middle-income countries. Hum Resour Health 2017;15:1–13.

[45] **Nyein PP**, **Tillakeratne S**, Phyu S, et al. Evaluation of simplified HCV diagnostics in HIV/HCV co-infected patients in Myanmar. Viruses 2023;15(2):521.
[46] Leidner AJ, Chesson HW, Xu F, et al. Cost-effectiveness of hepatitis C treatment for patients in early stages of liver disease. Hepatology 2015;61(6):1860–1869.
[47] NSW Government. NSW hepatitis C annual data report. 2023. Australia.
[48] Liver Foundation. The social and economic cost of primary liver cancer hepatocellular carcinoma (HCC) in Australia. 2021. Australia.
[49] Shah SHBU, Alavi M, Hajarizadeh B, et al. Liver-related mortality among people with hepatitis B and C: Evaluation of definitions based on linked healthcare administrative datasets. J Viral Hepat 2023;30(6):520–529.
[50] Blach S, Kondili LA, Aghemo A, et al. Impact of COVID-19 on global HCV elimination efforts. J Hepatol 2021;74(1):31–36.

Keywords: HCV; late diagnosis; late treatment; DC; HCC; end-stage liver disease; decompensated cirrhosis; hepatocellular carcinoma; DAA.
Received 10 February 2025; received in revised form 27 May 2025; accepted 29 May 2025; Available online 6 June 2025

Journal of Hepatology, Volume *7*

Supplemental information

Missed opportunities in HCV care: Trends in late diagnosis and treatment

Shane Tillakeratne, Heather Valerio, Maryam Alavi, Behzad Hajarizadeh, Marianne Martinello, Kathy Petoumenos, Jacob George, Janaki Amin, Gail V. Matthews, Jason Grebely, Sallie-Anne Pearson, and Gregory J. Dore

Missed opportunities in HCV care: Trends in late diagnosis and treatment

Shane Tillakeratne, Heather Valerio, Maryam Alavi, Behzad Hajarizadeh, Marianne Martinello, Kathy Petoumenos, Jacob George, Janaki Amin, Gail V. Matthews, Jason Grebely, Sallie-Anne Pearson, Gregory J. Dore

Table of contents

Table S1……………………………………………………………………………2

Table S2……………………………………………………………………………3

Table S3……………………………………………………………………………5

Fig. S1……………………………………………………………………………...6

Table S4……………………………………………………………………………7

Table S5……………………………………………………………………………8

Table S1. Set of relevant ICD-10 codes for decompensated cirrhosis (DC) and hepatocellular carcinoma (HCC) diagnoses

Inferred Diagnosis	ICD-10 Code	First-time hospitalisation discharge diagnosis
Decompensated Cirrhosis (DC)	R18	Ascites
	I85.0	Oesophageal varices
	I98.3	Oesophageal varices with bleeding in diseases classified elsewhere
	K72.1	Chronic hepatic failure
	K72.9	Hepatic failure, unspecified
	K70.4	Alcoholic hepatic failure
	K76.7	Hepatorenal syndrome
Hepatocellular carcinoma (HCC)	C22.0	Liver cell carcinoma

\

Table S2. ICD-10 definitions used to identify injecting drug use-related hospital presentations among all NSW people with an HCV notification and ESLD diagnosis, NSW 2010-2021

ICD-10	Description
A40	Streptococcal sepsis
A41	Other sepsis
A48.0	Other bacterial diseases, not elsewhere classified (gas gangrene)
B37.6	Candidiasis, candida endocarditis
F11	Mental and behavioural disorders due to use of opioids
F13	Mental and behavioural disorders due to sedatives or hypnotics
F14	Mental and behavioural disorders due to use of cocaine
F15	Mental and behavioural disorders due to use of other stimulants, including caffeine
F19	Mental and behavioural disorders due to multiple drug use and use of other psychoactive substances
G06	Intracranial and intraspinal abscess and granuloma
G09	Sequelae of inflammatory disease of central nervous system
I26.9	Pulmonary embolism, pulmonary embolism without mention of acute or pulmonale
I33	Acute and subacute endocarditis
I34	Nonrheumatic mitral valve disorders
I35	Nonrheumatic aortic valve disorders
I36	Nonrheumatic tricuspid valve disorders
I37	Pulmonary valve disorders
I38	Endocarditis, valve unspecified
I39	Endocarditis and heart valve disorders in diseases classified elsewhere
I40.0	Acute myocarditis, infective myocarditis
I80	Phlebitis and thrombophlebitis
K63.0	Other diseases of the intestine, abscess of intestine
K65.0	Peritonitis, acute peritonitis
K75.0	Other inflammatory liver disease, abscess of liver
L02	Cutaneous abscess, furuncle and carbuncle
L03	Cellulitis
L97	Ulcer of lower limb, not elsewhere classified
L98.8	Other disorders of skin and subcutaneous tissue, not elsewhere classified, other specified disorders of skin and subcutaneous tissue
M54.0	Dorsalgia, panniculitis affecting regions of neck and back
M72.6	Fibroblastic disorders, necrotizing fasciitis
M79.3	Other soft tissue disorders, not elsewhere classified (panniculitis, unspecified)
M86	Osteomyelitis
M89.9	Other disorders of bone, disorder of bone, unspecified
N10	Acute tubulo-interstitial nephritis
R02	Gangrene, not elsewhere classified
R57.2	Shock, not elsewhere classified, septic shock
R65.1	Systemic Inflammatory Response Syndrome of infectious origin with organ failure
R65.9	Systemic Inflammatory Response Syndrome, unspecified
R78.1	Finding of opiate drug in blood
R78.2	Finding of cocaine in blood
T38.7	Androgens and anabolic congeners
T40.0	Poisoning by narcotics and psychodysleptics, opium

T40.1	Poisoning by narcotics and psychodysleptics, heroin
T40.2	Poisoning by narcotics and psychodysleptics, other opioids (codeine/morphine)
T40.3	Poisoning by narcotics and psychodysleptics, methadone
T40.4	Poisoning by narcotics and psychodysleptics, other synthetic narcotics (pethidine)
T40.5	Poisoning by narcotics and psychodysleptics, cocaine
T40.6	Poisoning by narcotics and psychodysleptics, other and unspecified narcotics
T40.8	Poisoning by narcotics and psychodysleptics, lysergide (LSD)
T41.2	Poisoning by anaesthetics and therapeutic gases, other and unspecified general anaesthetics
T42.3	Poisoning by antiepileptic, sedative-hypnotic and antiparkinsonism drugs, barbiturates
T42.4	Poisoning by antiepileptic, sedative-hypnotic and antiparkinsonism drugs, benzodiazepines
T42.5	Poisoning by antiepileptic, sedative-hypnotic and antiparkinsonism drugs, mixed antiepileptics, not elsewhere classified
T42.6	Poisoning by antiepileptic, sedative-hypnotic and antiparkinsonism drugs, other antiepileptic and sedative-hypnotic drugs
T42.7	Poisoning by antiepileptic, sedative-hypnotic and antiparkinsonism drugs, antiepileptic and sedative-hypnotic drugs, unspecified
T42.8	Poisoning by antiepileptic, sedative-hypnotic and antiparkinsonism drugs, antiparkinsonism drugs and other central muscle-tone depressants
T43.6	Poisoning by psychotropic drugs, not elsewhere classified, psychostimulants with abuse potential
T43.8	Poisoning by psychotropic drugs, not elsewhere classified, other psychotropic drugs, not elsewhere classified
T43.9	Poisoning by psychotropic drugs, not elsewhere classified, psychotropic drug, unspecified
T50.7	Poisoning by psychotropic drugs, not elsewhere classified, analeptics and opioid receptor antagonists
X41	Accidental poisoning by and exposure to antiepileptic, sedative-hypnotic, antiparkinsonism and psychotropic drugs, not elsewhere classified
X61	Intentional self-poisoning by and exposure to antiepileptic, sedative-hypnotic, antiparkinsonism and psychotropic drugs, not elsewhere classified
Y11	Poisoning by and exposure to antiepileptic, sedative-hypnotic, antiparkinsonism and psychotropic drugs, not elsewhere classified, undetermined intent

Table S3. ICD-10 definitions used to identify alcohol-use disorder hospital presentations among all NSW people with an HCV notification, NSW 2010-2021

Inferred Diagnosis	ICD-10 Code	Alcohol-use disorder-related hospital admission
**Alcohol-use disorder**	E24.4	Alcohol induced Pseudo-Cushing's syndrome
	F10	Mental and behavioural disorders due to use of alcohol
	G31.2	Degeneration of nervous system due to alcohol
	G62.1	Alcoholic polyneuropathy
	I42.6	Alcoholic cardiomyopathy
	G72.1	Alcoholic myopathy
	Z50.2	Alcohol rehabilitation
	Z71.4	Alcohol abuse counselling and surveillance

Fig. S1. Trends in HCV diagnosis timeliness among people with (A) DC and (B) HCC diagnosis, 2010-2021.

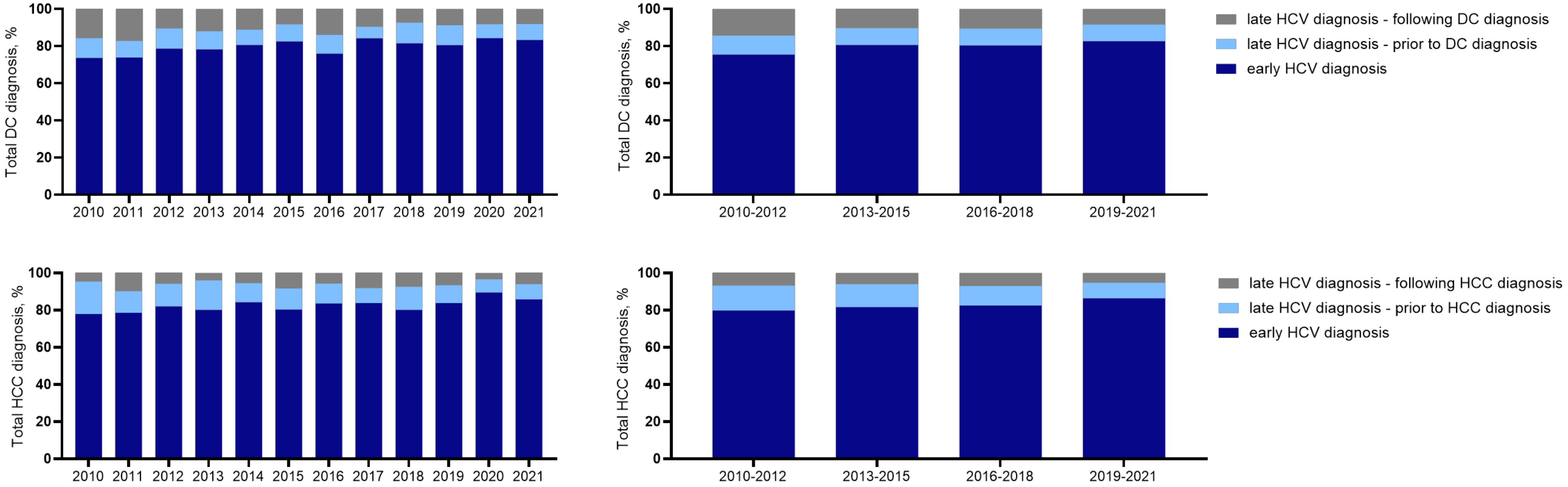

People with an HCV notification and ESLD diagnosis (n = 4,419). (A) People with an DC diagnosis (n = 2,580). (B) People with an HCC diagnosis (n = 1,839). People with an HCV notification and DC/HCC diagnosis (n = 4,419). Late HCV diagnosis – following (within a month or after DC/HCC diagnosis); late HCV diagnosis – prior (0-2 years prior to DC/HCC diagnosis); early HCV diagnosis (>2 years prior to DC/HCC diagnosis). Levels of significance: $p < 0.001$ (Chi-squared test).

Table S4. Proportion of treatment timeliness among people with an HCV diagnosis and ESLD, by time period.

Time period	Total	No Treatment	%	Late treatment	%	Early treatment	%
2010-2021	4,419	2,870	65	1,111	25	438	10
2010-2012	1,081	915	85	148	13	18	2
2013-2015	1,185	837	71	311	26	37	3
2016-2018	1,160	639	55	436	38	85	7
2019-2021	993	479	48	216	22	298	30

Table S5. Demographic characteristics among NSW people with an HCV diagnosis and ESLD by treatment timeliness, 2016-2018 and 2019-2021.

Characteristics, n (row%)	2016-2018						2019-2021					
	**No treatment**	**%**	**Late treatment**	**%**	**Early treatment**	**%**	**No treatment**	**%**	**Late treatment**	**%**	**Early treatment**	**%**
Total, n (row%)	639	55	436	38	85	7	479	48	216	22	298	30
Birth cohort												
≤1944	49	71	19	27	-		36	67	5	9	13	24
1945-1959	268	52	194	38	49	10	185	48	65	17	134	35
1960-1974	269	55	189	39	30	6	217	46	123	26	132	28
≥1975	53	58	34	37	-		37	47	23	29	19	24
Sex [a]												
Male	478	54	344	39	61	7	345	47	169	23	217	30
Female	160	59	92	34	18	7	131	51	47	18	81	31
Region of HCV notification[a]												
Metropolitan	134	51	107	41	23	8	125	55	39	17	60	28
Outer metropolitan	247	60	134	33	29	7	156	48	70	21	101	31
Rural/Regional	244	53	184	40	31	7	183	45	99	24	124	31
Country of birth[a]												
Australia	512	53	383	40	69	7	401	48	182	22	253	30
Overseas	127	65	53	27	16	8	78	50	34	22	45	28
Injecting drug use												
No history	215	53	148	37	40	10	188	49	86	22	113	29
Distant	85	52	69	42	9	6	64	47	25	19	46	34
Recent	339	57	219	37	36	6	227	48	105	22	139	30
Opioid agonist therapy												
No history	468	54	327	38	73	8	370	50	159	17	216	33
Distant	110	57	75	39	7	4	74	45	34	20	58	35
Recent	61	61	34	34	5	5	35	43	23	28	24	29
Alcohol use disorder												
No history	269	56	166	35	45	9	209	47	86	19	152	34
Distant	53	56	32	34	10	10	38	47	11	14	32	39
Recent	317	54	238	41	30	5	232	50	119	26	114	24
Incarceration												
No history	477	55	328	38	65	7	347	50	142	20	215	30
Distant	117	56	78	37	15	7	112	49	56	24	63	27
Recent	45	56	30	38	5	6	20	35	18	31	20	34
Government assistance												
No	60	54	42	38	9	8	45	54	14	17	24	29
Distant	68	46	63	43	17	11	56	43	34	26	40	31
Recent	511	57	331	37	59	6	378	49	168	22	234	29

[a]Among people with available information. HCV, hepatitis C virus; NSW, New South Wales. Small cells (cell count with values <5) were suppressed in this analysis.
